# Quantum Molecular Resonance Electrical Stimulation as a Beneficial and Safe Treatment for Multifactorial Dry Eye Disease

**DOI:** 10.7759/cureus.39695

**Published:** 2023-05-30

**Authors:** Dimitra Kavroulaki, Elena Konstantinidou, Anastasia Tsiogka, Konstantinos Rallis, Emmanuel Mavrikakis

**Affiliations:** 1 Ophthalmology, General Hospital of Athens G. Gennimatas, Athens, GRC; 2 Opthalmology, General Hospital of Athens G. Gennimatas, Athens, GRC

**Keywords:** ocular surface disease, meibomian gland disease, quantum molecular resonance, transcutaneous electrical stimulation, dry eye

## Abstract

Introduction: To assess the clinical benefits obtained with transcutaneous low-power, high-frequency quantum molecular resonance (QMR) electrotherapy in a group of multifactorial dry eye patients.

Methods: Fifty-one patients (total of 102 eyes) with dry eye symptoms were enrolled in the study. Included clinical conditions were meibomian gland dysfunction, glaucoma, cataract surgery within the past six months, and autoimmune disease-related superficial punctuate keratitis. The QMR treatment was administered using the Rexon-Eye device (Resono Ophthalmic, Sandrigo, Italy) for four consecutive weeks, with one 20-minute treatment session per week. The measured ocular parameters included non-invasive tear break-up time (NIBUT), corneal interferometry, lower eyelid meibography, and tear meniscus height, all measured at baseline, at the end of treatment, and two months after the end of treatment. The Ocular Surface Disease Index (OSDI) questionnaire was gathered at the same time. The study has received approval from our institution’s ethics committee.

Results: At the end of treatment, interferometry, tear meniscus height, and OSDI score improved at a statistically significant level. No statistically significant change was observed in NIBUT or meibography. At two months after the end of treatment, all parameters showed a statistically significant improvement, namely NIBUT, meibography, interferometry, tear meniscus, and OSDI score. No adverse events or side effects were reported.

Conclusions: The QMR electrotherapy by the Rexon-Eye device shows statistically significant improvement of dry eye clinical signs and symptoms with a duration of at least two months.

## Introduction

The significant increment at all ages of symptoms of dry eye disease (DED), due to air pollution, overuse of beauty products, and excessive screen time, has prompted the development of new, more effective treatment strategies for dry eye. A recent promising approach is electrical stimulation based on quantum molecular resonance (QMR). This innovative technology is based on the transcutaneous delivery of low-intensity, high-frequency alternating electrical current producing high-frequency non-ionizing waves (4 MHz to 64 MHz). The electromagnetic fields produced have been shown to be capable of stimulating the metabolism and natural regeneration of biological tissue and cells. It is possible to explain this effect based on various phenomena generated by the QMR stimulation and experimentally observed: mechanical deformation of the cell membrane, increase in calcium release, and activation of signaling pathways [1]. Using sophisticated microarray techniques to evaluate gene expression, a more recent in vitro study on mesenchymal stromal cells has shown that QMR is able to up-regulate genes involved in extracellular matrix (ECM) remodeling, embryogenesis, wound healing, and angiogenesis [2]. This technology has been shown to provide excellent results in wound healing in human patients [3] and, more recently, in animal studies related to very severe corneal inflammation [4]. QMR was then applied with the Rexon-Eye device (Resono Ophthalmic, Sandrigo, Italy) in the treatment of DED [5]. Several studies have provided evidence that it stimulates and reactivates all aspects of the lacrimal system, improving tear and lipid secretion and providing significant benefits to patients [5-8]. The aim of this study is to assess the effect of this treatment in a cohort of patients suffering from multifactorial dry eye disease, including meibomian gland dysfunction, cataract surgery, glaucoma, and keratitis related to autoimmune disease.

## Materials and methods

Fifty-one patients (102 eyes) randomly selected from the outpatient clinic of our tertiary hospital were enrolled in the study. The median age was 57.4 years ± 15.67 (SD) (range, 21 to 84 years), 12 (23.53%) were male, and 39 (76.47%) were female. Inclusion criteria were dry eye symptoms with a value higher than 18 as quantified by the Ocular Surface Disease Index (QSDI) questionnaire, caused by meibomian gland dysfunction (27 patients), glaucoma (two patients), cataract surgery within the past six months (four patients), and superficial punctuate keratitis related to autoimmune disease, specifically primary Sjogren, thyroid eye disease, and rheumatoid arthritis (18 patients). Treatment was performed with the Rexon-Eye device, which consists of a generator transmitting a low-power, high-frequency alternating electrical current to a specially designed mask containing two electrodes, one for each eye. The mask was placed over the patient’s closed eyelids, and the current was applied to the eyelids and periocular skin for 20 minutes, with a 30-second alternation between eyes. The session was repeated once per week for a total of four consecutive weeks, according to manufacturer guidelines. The IDRA device (SBM Sistemi, Turin, Italy) was used to objectively quantify dry eye ocular parameters, specifically non-invasive tear break-up time (NIBUT), corneal interferometry, lower eyelid meibography, and tear meniscus height. This ocular surface analyzer provides a personalized measurement of the three layers of the tear film and the meibomian glands of the upper and lower eyelids. Interferometry evaluates the lipid layer thickness, the tear meniscus, and the thickness of the tears on the eyelid margin, providing information on the tear volume. NIBUT evaluates the stability of the mucin layer between blinking and meibography, which is the visualization of the glands through the illumination of the eyelid with infrared light, measuring meibomian gland loss. All ocular parameters were measured before the initiation of treatment (baseline), at the end of the fourth and last session, and two months after the end of treatment. Patients’ satisfaction was recorded at the same time with the Speed II Ocular Surface Disease Index questionnaire. Subjective evaluation of symptoms and satisfaction was documented on a scale from zero to four and was all added up to a final number. The study has received approval from our institution’s ethics committee. Informed consent was obtained from all patients. Recruitment of patients, treatment protocols, and measurements were conducted by the same physicians. All 51 patients (a total of 102 eyes) were analyzed as one group. At the two-month follow-up, only 32 patients managed to attend. Data analysis was performed with STATA version 13 (StataCorp LLC, Texas, USA), comparisons of score values were performed with the paired t-test for normally distributed differences, and all reported p-values were based on two-sided tests and compared to a significance level of 5%.

## Results

**Figure 1:**
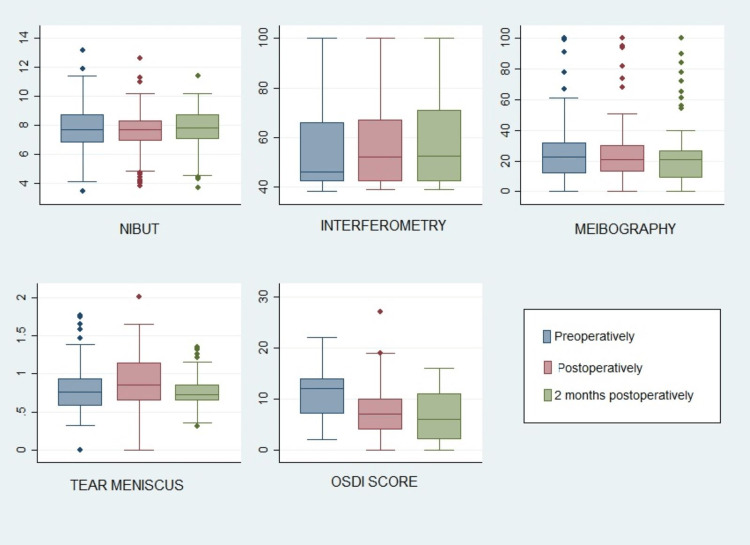
Box plots of each examined variable baseline (pre-operatively), end of treatment (post-operatively), and two months after the end of treatment (two months post-operatively)

At the end of the treatment (EoT), interferometry (p=0.021), tear meniscus height (p = 0.002), and OSDI score (p<0.001) improved to a statistically significant level compared to baseline values. There was no statistically significant change in the values of NIBUT (p=0.891) and lower eyelid meibography (p=0.962). At the two-month follow-up, a total of 32 patients were analyzed. With respect to baseline, NIBUT showed a statistically significant increase (p = 0.002), and lower eyelid meibography showed a statistically significant decrease in glands loss (p=0.003) when compared to baseline values. The scores remained significantly improved in the interferometry values (p<0.001), tear meniscus (p<0.001), and OSDI scores (p<0.001). No adverse events or side effects were recorded. No thermal or electrical burns were noted (Table 1 and Figure 1).

**Table 1:** Statistical analysis of results at baseline, end of treatment, and two months after the end of treatment EoT: end of treatment, NIBUT: non-invasive tear break-up-time, OSDI: Ocular Surface Disease Index

	NIBUT	Interferometry	Meibography	Tear meniscus	OSDI score
Baseline	7.50± 0.18	53.32± 1.8	25.28±2.57	0.81±0.04	10.83±0.57
EoT	7.53±0.19	58.13±2.2	25.46±2.52	0.94±0.04	7.63±0.62
p-value	0.891	0.021	0.962	0.002	<0.001
Two months after EoT	7.59±0.18	59.77±2.60	23.98±2.89	0.74±0.04	6.38±0.63
p-value	0.002	<0.001	0.003	<0.001	<0.001

## Discussion

This larger-scale study supports and confirms the safety and efficacy of the QMR technology in the treatment of DED, as shown in previous studies [5-8] and also in our cohort of multifactorial dry eye patients. The different etiology of the dry eye signs and symptoms in our patients might have hampered the outcome of the treatment, but on the other hand, it shows promising results not only in patients with meibomian gland dysfunction but also in patients with autoimmune diseases like rheumatoid arthritis, primary Sjogren syndrome, and thyroid eye disease. All objectively measured ocular parameters showed significant improvement post-treatment, as did patients’ subjective symptoms of irritation, itching, ocular redness, and discomfort for a period of two months. The treatment appears to reactivate the lacrimal system, supposedly through the regeneration of goblet and lacrimal gland cells, improving tear secretion early in the course of treatment. Meibomian glands appear to regenerate later in time with an increase in their number, affecting lipid layer secretion and tear break-up time around two months post-therapy. There are few studies related to electrotherapy for DED. The study by Cai and Zhang [9] tested the efficacy and safety of the combination of transcutaneous electrical stimulation, with a current at 20 Hz and therefore not based on the QMR effect, with artificial tears on 44 eyes. The electrodes were placed on the periorbital skin, and treatment was administered for 20 sessions (five sessions per week for four weeks). They reported a statistically significant improvement in OSDI scores, tear break up time (TBUT), Schirmer’s I test, and corneal fluorescein staining at four weeks after treatment, with no adverse events. As regards the studies specifically related to QMR electrotherapy in DED, Pedrotti et al. [5] applied periorbital stimulation using skin electrodes and manually with a handpiece conductor moved by the operator in 12 sessions over a period of two months. They reported statistically significant improvements in OSDI scores, TBUT, Schirmer’s I test, and fluorescein staining at the six- and twelve-month follow-ups, with a reduced need for artificial tears. Ferrari et al. [6] tested the effects of the Rexon-Eye treatment on 25 patients with meibomian gland dysfunction, showing significant reductions in all the signs and symptoms of DED associated with gland dysfunction taken into account in their study. Trivli et al. [7] have applied QMR technology to the treatment of mixed-type DED, with remarkable improvements in signs and symptoms, in particular as regards lipid layer composition and corneal inflammation. More recently, Shemer et al. [8] presented the preliminary results obtained in a subset of patients from their randomized controlled trial with the Rexon-Eye, where significant improvements were obtained in OSDI, meibomian gland disease score, and corneal staining in treated subjects versus controls.

## Conclusions

This study has confirmed in our cohort of multifactorial DED patients the significant improvement of dry eye clinical signs and symptoms observed in other clinical studies employing QMR electrotherapy with the Rexon-Eye device with a duration of at least two months. Further investigations with a longer follow-up period may be needed to fully elucidate the expected duration of the benefits for the patients and the improved range of clinical conditions.
